# Gendered risks: access to and utilization of sexual and reproductive health services among young migrants in Southwestern Uganda: the role of the ‘lending a hand’ intervention

**DOI:** 10.3389/frph.2024.1256485

**Published:** 2024-05-06

**Authors:** Rachel Kawuma, Edward Tumwesige, Allen Asiimwe, Sarah Bernays, Janet Seeley

**Affiliations:** ^1^ MRC/UVRI & LSHTM Uganda Research Unit, Department of Social Sciences, Entebbe, Uganda; ^2^ School of Public Health, University of Sydney, Sydney, NSW, Australia; ^3^ Department of Global Health and Development, London School of Hygiene and Tropical Medicine, London, United Kingdom; ^4^ Africa Health Research Institute, KwaZulu-Natal, South Africa

**Keywords:** migration, sexual and reproductive health, gender, adolescents and young people, Uganda

## Abstract

**Introduction:**

Young migrants may engage in risky behaviours due to social, economic, and psychological challenges as they try to “get by” in their new host communities. This can result in unintended pregnancies, sexually transmitted infections including HIV, and poor mental health outcomes.

During a study to test the feasibility and acceptability of an early intervention to reduce the harm of patterns of risk associated with migration, we assessed access to and utilization of sexual and reproductive health services (SRH) among recent migrants (14–24 years) in south-western Uganda.

**Methods:**

The intervention conducted in 2022/23 involved training peer supporters to provide referral advice and support to young people. Between March–November 2022, 20 young migrants (11 males and 9 females) were purposively selected to participate in two in-depth interviews each to explore their experiences during the intervention. Data were analysed thematically.

**Results:**

Women engaged in transactional sex to supplement their low pay while men got involved in risky behaviour once they had earned some money. Many suffered from sexually transmitted infections-related symptoms, were at risk for HIV infection and some women had fears of unwanted pregnancy. While some tried to seek for SRH services from public facilities, poor health service delivery such as long queues and shortages of drugs, discouraged them from going there. Young people tried to access treatment from private facilities but could not afford the costs. The intervention increased knowledge about SRH and supported young people to access services from the public health facilities at no cost thus increasing utilization.

**Conclusion:**

Sexual health risks were experienced differently by women and men. The women were likely to experience symptoms related to sexually transmitted infections (including HIV) much earlier than men and this could increase their likelihood to engage with SRH services. The intervention served to increase men's readiness to access SRH services by providing them at a time and place that is convenient. Recognizing the different risk profiles of young people is important in tailoring appropriate interventions to promote equitable access and utilization of SRH services for both genders in this vulnerable population.

## Introduction

Migration of people from rural to urban areas has implications for public health including access to and utilization of sexual and reproductive health (SRH) services. In Africa, the number of people who migrate from rural to urban areas keeps increasing with adolescents and young people (AYP) among those moving to urban areas (1, 2). Young people move in search of opportunities for education and work to earn to support themselves and the people they leave behind (3, 4). However, as they settle and try to get by in their new host communities, young migrants face social, economic, and psychological challenges (5) which may force them to engage in risky lifestyles such as providing transactional sex to get access to support and using drugs and alcohol (6, 7). This may result in unintended pregnancies, sexually transmitted infections (STIs) including HIV, and poor mental health outcomes.

The United Nations 2030 Agenda for Sustainable Development, “Leave No One Behind” encourages governments to integrate the health needs of migrants into national plans, policies, and strategies across sectors (8). Researchers have noted low utilization of SRH services among migrants compared to non-migrants (9, 10). Poorer SRH outcomes have been recorded among migrant girls and women compared to the men (11, 12). In particular, young migrants have challenges navigating the health care landscape partly due to lack of familial support and sometimes language barriers (13) and stigma attached to seeking the services (14).

The barriers to accessing and using SRH services for migrants have been identified in many different settings. Ha et al. (15) who conducted research in Vietnam identified inconvenient clinic timings, cost and distance to the services as barriers to service use. Bwambale et al. (10) in their work with street children and young adults in Kampala note that a lack of information about where to seek need for SRH services affected younger migrants’ likelihood to seek care. Being denied access to health services and negative interactions with health care workers have also been identified as factors affecting migrants’ access and utilisation of SRH services in Africa and elsewhere (9, 16).

We set out to design and test an intervention that can improve access to and utilization of SRH services among young migrants. Between 2017 and 2019, formative research was conducted in South Africa and Uganda to investigate the experiences of young people moving to small towns from rural settings in relation to HIV risk, prevention and treatment seeking behaviour (17–19). As one of the outputs from this study, the young people contributed to suggestions to a co-designed intervention, which was later named “Lending a Hand”, aimed at tackling their needs. For instance, they suggested putting up structures with people they could trust to provide advice and support to migrants.

As follow up, a pilot study was conducted (January 2020–March 2023) to test the acceptability and feasibility of the “lending a hand” intervention among young migrants aged 14–24 years in two small towns in southwestern Uganda. In this paper, we use a gendered lens to assess the sexual health risks and needs of young women and men to reduce harm from the risks they face as new migrants in these communities.

## Methods

### Study design and setting

We conducted a qualitative methods study within an intervention aimed at reducing the harm of patterns of risk associated with youth migration among young people (aged 14–24 years) who had recently migrated within a period of not more than 9 months. Based on our previous work, we were aware that after 9 months young people had either already moved on to a new place or were settled enough in their new communities to share their experience from their first months. Between March 2022 and January 2023, a total of 385 young migrants were recruited into the intervention from two small towns found in Kalungu district, southwestern Uganda.

One of the towns (A) is located on the main transnational highway to neighbouring countries like Rwanda and Democratic republic of Congo (17, 20) and provides a popular stop over for long distance trucks mostly moving from Mombasa port to these countries. People of various backgrounds from within Uganda and neighbouring countries move to this town. In the last five years a large rice factory has been set up which attracts many young people seeking employment. Within the neighbourhood of this town there is a fishing community which also provides jobs for young people. The second town (B) is smaller. It is in a rural setting and attracts people from neighbouring villages looking for work, some planning to transition to bigger towns stop there with some staying for weeks, months or even years. According to the national population census of 2014, population estimates in the two towns were approximately 20,000 and 5,000 for town A and B respectively (21).

### Implementing the “lending a hand intervention”

The aim of this study was to develop and test the acceptability and feasibility of a protective support structure named “Lending a hand”. This was not intended to be a full intervention trial, this study was looking at feasibility and acceptability and assessing the components of the intervention.

Before implementing the intervention, a cross-sectional survey was conducted (November 2020–March 2021) among a section of young people (14–24 years) who had recently migrated to these places within the last 9 months, documenting their period of stay and their occupations. Some migrants who participated in the formative study in 2017–2019 supported the study team to recruit for the survey. All the young people who participated in the survey and were willing to take part in the intervention were encouraged to join.

Recruitment of young migrants was done through five young people (3 males and 2 females), also previous migrants but settled in these towns who were recruited as peer supporters to support the delivery of the intervention. They were trained in lay counselling, basic research, community mobilization and engagement, and referral skills so they would recruit young migrants from their communities, support them where they could, and/ or connect them to the study team (counsellor) or refer them to public health facilities. Each peer supporter was advised to approach and recruit into the intervention any young migrant, regardless of their gender.

The study team also conducted community mobilization and publicized the intervention in the area using the community radio, mobile vans, and posters.

Due to interruptions because of the extended COVID-19 lockdowns in Uganda (March–September 2020), which restricted face to face interactions, the study design of the intervention was modified from purely meeting the young people physically to establishing a toll-free telephone line that was used to offer support for young people in the intervention. This toll-free number was shared during the mobilisation drives with young migrants, so that they were aware that they could call and speak to a member of the study team. Those who called in and needed referral for medical services were directed to government health facilities with which we established a memorandum of understanding to support the young migrants. The phases of the study are shown Table 1 below.

**Table 1:** Implementation of the ‘lending a hand’ intervention among young migrants (14–24 years) between January 2020–March 2023.

Pre-intervention phase 2017–2019	• Formative research to investigate the experiences of young people moving to a town from a rural setting in South Africa and Uganda • To co-design an intervention with young people aimed at addressing their specific needs.
Baseline Phase January 2020–April 2021	• Community engagement activities (January–March 2020) • Preparing for IRB approvals • Cross sectional survey (November 2020–March 2021)
Preparation phase May 2021–October 2021	• Recruit project staff – Identification/recruitment of Peer supporters – Training of Peer supporters – Established MoU with health centres in town A and B • Establish a call center
Implementation Phase October 2021–January 2023	• Participant recruitment/enrolment – Referral to health centres in Town A and B – Call centre – Qualitative data collection phase (March–November 2022) • Qualitative data analysis (September 2022–January 2023) • Study dissemination

### Conceptual framework and theory of change

**Figure 1:**
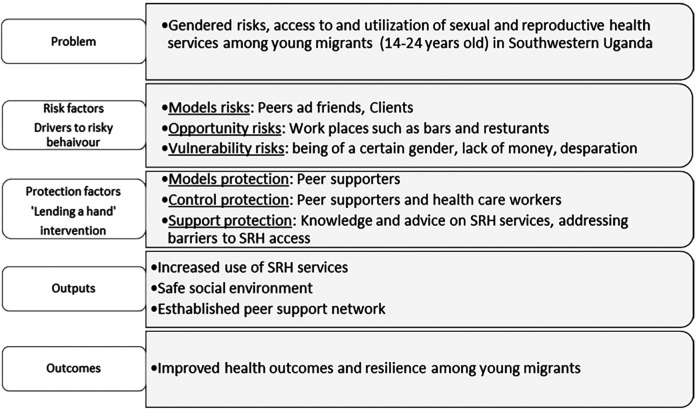
Theory of change adapted from the protection-risk framework by Kabiru et al. (23).

The intervention structure, designed with the young people, was then mapped on to the protection-risk framework (22) to produce a theory of Change to guide the evaluation of the project. This framework highlights three categories of risk factors namely: models risk, opportunity risk, and vulnerability risk and three categories of protective factors: models protection, controls protection and support protection. Specifically, in this intervention risk factors are those that are likely to drive young people to problem behaviour such as excessive alcohol drinking, use of recreational drugs and risky sexual behaviour, while protective factors are those that mediate to decrease the likelihood of engaging in the problem behaviour (Figure 1).

### Sampling and recruitment for the qualitative study

Twenty young people (both males and females) were purposively selected from those participating in the intervention to take part in two in-depth interviews (IDI) each, between March and November 2022. Sampling characteristics included age, and the work type they were engaged in. They were recruited from areas such as sex work locations, bars and lodges, roadside markets, the rice factory and skills training centres like motor vehicle repairs garages and hair salons.

### Data collection

We intended to conduct two IDIs with each participant, one within the first month and the second after six months of being part of the intervention and indeed for the first IDI, we interviewed all the 20 young people (11 males and 9 females). However, we only managed to conduct a second interview with 14 (10 males and 4 females) because the rest had moved away from these towns, and we could not contact them physically (Table 2) to have an interview with them. The reason for conducting a second interview was to capture changes in their lived experiences over time.

**Table 2:** Characteristics of study participants in the “lending a hand” study qualitative study conducted between March–November 2022 in Uganda.

ID	Gender	Age	Work	Relationship type	Education	IDI-1	IDI-2	Reason no IDI-2
P01	Female	22	Bar and restaurant	Single	Never went to school	Yes	Yes	
P02	Male	20	Multiple jobs	Single	Secondary	Yes	Yes	
P03	Male	16	Unemployed (Student)	Single	Primary	Yes		Busy, not interested
P04	Male	22	Other (fishing)	Single	Primary	Yes	Yes	
P05	Male	18	Multiple jobs	Married	Secondary	Yes	Yes	
P06	Female	22	Unemployed (“housewife”)	Married	Primary	Yes		Migrated, no contact
P07	Male	22	Rice Farm	Single	Primary	Yes	Yes	
P08	Male	23	Unemployed	Single	Secondary	Yes	Yes	
P09	Female	22	Unemployed (student)	Single	Secondary	Yes		Migrated after training
P10	Female	18	Other (shop attendant)	Single	Secondary	Yes		Migrated, no contact
P11	Female	17	Bar and restaurant	Single	Secondary	Yes		Migrated, no contact
P12	Female	20	Multiple jobs	Single	Secondary	Yes	Yes	
P13	Female	20	Bar and restaurant	Single	Primary	Yes		Re-located back home
P14	Male	19	Multiple jobs	Single	Secondary	Yes	Yes	
P15	Female	20	Multiple jobs	Single	Secondary	Yes	Yes	
P16	Male	18	Multiple jobs	Single	Primary	Yes	Yes	
P17	Female	17	Bar and restaurant	Single	Secondary	Yes	Yes	
P18	Male	21	Rice Farm	Married	Secondary+	Yes	Yes	
P19	Male	21	Rice Farm	Single	Primary	Yes	Yes	
P20	Male	21	Other	Married	Secondary+	Yes	Yes	

During the first set of IDIs, conducted from March to April 2022, we collected information on life history, mobility patterns and experiences of settling down in the new communities, their access and use of SRH services and experiences during the COVID-19 lock downs. In the second set of IDIs, carried out from August to November 2022, we probed further about their life history, influences on risk taking, and their experience with and recommendations they had about the intervention.

Recruitment into the study and all IDIs were conducted by two researchers, male and female, co-authors ET and AA, both of whom are close in age to the young people. The two researchers also had experience interacting with young people having conducted the previous phase of the study. All IDIs were conducted using a topic guide, in a language that was preferred by the participants and took place in private places often suggested by participants. Interviews lasted between 45 and 60 min and were audio recorded after obtaining consent. Brief notes were written down to pick up details such as the non-verbal expressions which could not be recorded. De-briefing meetings were held regularly between interviewers and the study team (the other co-authors of this paper) to ensure completeness of the data, and to identify gaps identified in the data collected which were filled during subsequent interviews.

### Data management and analysis

All data collected were de-identified by using numbers in place of participant names. The audio recordings were backed up on secure servers, only accessed by the study team. Information was transcribed directly into English by the interviewers before transcripts were coded. The analysis process was conducted following the principles of thematic analysis (24) which started by getting familiar with what was coming out of the data, often discussed during the debriefing meetings which helped us identify similar patterns of lived experiences by either female and male migrants. Then the two researchers who collected the data, initially read two identical scripts and each identified common codes which guided formation of a final coding framework that was used to code the rest of the scripts. Emerging codes with similar meaning were combined to create themes, and analytical memos on those themes were produced by the two researchers and discussed with the wider team. These analytical memos formed the building blocks for the results section of this paper.

In order to cross-check the trustworthiness of our emerging findings, we held sharing sessions with the peer supporters to discuss the results. We also held a sharing event with participants at the end of the study where we presented the findings and received their feedback and comments on what we were planning to report.

## Results

### Characteristics of participants

The 20 young people were aged between 16 and 23 years and of these, 9 were females and 11 males. Twelve had reached secondary level, with two attaining a certificate following further training, one in electrical installation and another in tourism and travel. Four of the females worked in bars and restaurants, while the males usually held more than one job in construction, roadside vending and loading vehicles in markets or metal work. Others were engaged in fishing and serving in shops. Three people (2 students and I housewife) were categorised as unemployed (see Table 2 below).

Three broad themes emerged following analysis; first, we describe the drivers of the risks faced by the young people, specifically risky behaviour among them, secondly, we assess the barriers to seeking SRH care and treatment and lastly, we examine how the lending a hand intervention was able to engage in redressing and/or mitigating these specific risks.

### Drivers to SRH risks among these young migrants

For all the young people, the desire to work and be able to earn an income was important as they settled into the new communities. Many got unskilled manual jobs and often earned very little pay, so it was common to have more than one piece of work. For instance, some of the women worked in bars and restaurants, and did laundry work to supplement their income as narrated by one 17-year-old woman:

*“I searched for work and fortunately got a job in a restaurant where I work now and luckily am given free food (both lunch and supper) and paid three thousand shillings (<1$) daily. In addition, people call me to go and wash for them for which they pay me five or three thousand shillings depending on the volume of clothes they have that are to be washed”.*

The men worked as casual labourers in the roadside markets, construction sites and in nearby farms and gardens. One 19 years old male said:

*“I do a variety of jobs that are available to me for example, helping people in their gardens, going to the market, and carrying luggage for people, as well as portering at the construction sites. That is how I generally earn a living and is usually very little money which doesn’t exceed five thousand shillings (>1 $).”*

Earning very little income was the main driver for engaging in risky sexual behaviour, particularly for some of the women who got involved in transactional sex to supplement their income. Many got boyfriends who would provide support in exchange for sex, or they sold sex either at demarcated physical spaces or with people they interacted with at the workplaces, in bars and restaurants. One female (22 years old) mentioned that:

*“I am now so much into sex work because this is where I earn most of the money I survive on here because I do not have papers (academic documents) to get a job. The other jobs, for example working in a restaurant pay you three thousand shillings (<1$) for the whole day and this is not enough to pay rent for the room I sleep in” (Female, 22 years old).*

On the other hand, the men mostly became involved in risky behaviour due to peer influence. Once they were paid for the work done, several of them “congratulated” themselves for work well done by buying sex or drinking alcohol. While discussing some of the risks that young men face, one male (21 years) said this about his friends:

*“When they pay them, they come directly from the factory and head there in the corner to buy sex workers, and they go with them to party… Most of them use drugs like taking marijuana and booze, and their lives are not admirable. Another thing is that many have failed to do their work because they booze, you find that he is drunk for the whole night and spends the day sleeping yet they must work.”*

Another male (20 years old) discussed his personal experience, saying that after getting some money and renting his own room he invited over a girl for a night and had unprotected sex with her but, he said: “*I got so worried because of what I had done because I did not know the girl so well and I did not know whether she was okay (HIV negative)”*

### Barriers to seeking timely SRH care and treatment

As a result of the risky sexual behaviour, many young people suffered from STI related symptoms, and the women developed fears over unwanted pregnancy. They sought treatment from private facilities near their homes. However, both men and women could not afford the costs of treatment and therefore, they either paid for half or abandoned the treatment altogether like one female (20 years old) who narrated that even though she was in pain, she could not afford to pay all the money required to get the necessary treatment:

*“When I went to [mentions name of private health facility] to seek treatment, they asked me to pay twenty thousand shillings (*∼*4 $) [for a tube to clear the infection in her private parts], I left because I did not have the money, but they gave me some tablets that I swallowed which I had five thousand shillings (>1 $) for”.*

A few participants tried to seek treatment from known government aided health facilities but did not find the drugs they needed and instead were given a prescription to go and buy from pharmacies. As a result, the young people shunned those services:

*“Sometimes you find that they [public facilities] don’t have the drugs for the disease you would present with, and they just prescribe for you the drugs, so you go and buy from another place but remember, you do not have the money to buy those drugs. That is why most times you would rather go to the clinic than go to a government health centre.” (Male, 21 years old).*

### The role of the “lending a hand” intervention in mitigating these risks

The “lending a hand” intervention was delivered through peer supporters to provide knowledge and advice about SRH services such as STI treatment, HIV testing and counselling and prevention products like condoms and pre-exposure prophylaxis (PrEP) to migrant young people in the community. One young man disclosed that he learnt how to use condoms through his peer supporter:

‘*I even did not know how to use a condom, but I got all this information from her. I did not know that a condom has an expiry date, but I learnt this from her*’ (Male, 19 years old).

Not only did some of the young people gain knowledge about use of SRH prevention methods, but the intervention through the peer supporters also helped to provide information about existing SRH services to suit the needs of the young people and were supported to access them:

*“Through the peer supporters’ advice, I recently went to the health facility and got PrEP and even right now I have two packs of PrEP in my room. I take this medicine every day so that I don’t miss out on those men who don’t want to use any protection but are willing to give me some good money. For example, there is a man that comes and says that he is paying thirty thousand shillings [*∼*6.5$] for unprotected sex, and I accept because I need this money due to the current economic situation”. (Female, 22 years old)*

In addition, through a memorandum of understanding with some public facilities in the community, young people who were referred through the intervention were served by dedicated health care workers minimizing the time they spent at the facility. As a result, many were happy that they were able to receive treatment in this way:

*“The peer supporter took me to the health centre and the nurse gave me drugs and I got healed very well. I was so happy she helped me because I didn’t have money for treatment by that time… There were very many people lined up whom I found there but the nurse just called me to go directly to the check-up room. I was asked how I was feeling, they took my blood sample, I was given drugs and then we left the health centre premises” (Female, 17 years old)*

To some young migrants, the intervention helped them to deal with the stigma associated with being seen to access SRH services, so they liked the fact that they were able to get them in a discrete manner. For instance, one young man (20 years) noted that while condoms are freely available at the health facility, he was not comfortable picking them on his own:

*“This kind of arrangement has helped bring services nearer to us because sometimes you need condoms and going to the health facility to access them is not easy because we are always scared of people seeing us carrying condoms but when we come to the peer supporter, we get them so fast and she puts them in a small black polythene bag so that others don’t see what we are carrying. Even when she does not have them, she asks you to wait until she gets them the next day and brings them to your place”.*

The convenience of being able to access these products through their peer supporters made it possible even for those who stayed on at their jobs for long hours or working late in the night to access services. This was particularly useful for the young males as they did different jobs and did not have time during the day to seek care. A 19-year-old male commented that:

*“some of us are mobile so even if the health workers came to the community, we may not know that they have come. For example, early in the morning, I may be called to load sand on trucks in the quarries or I am busy in the garden but here she [peer supporter] is our friend and she understands our situation and she makes everything easy for us”.*

One 21-year-old male summed up the role of this intervention by saying:

*“Maybe we should have more people in the community like [mentions peer supporter] supporting young people because instead of buying medicine when I get sick, I run to her, and I save this money. Even if it is a big health condition, I know that she will support me to go to the hospital. Indeed, if you want to pass on information to young people and you go through [mentions peer supporter], you will get so many of them because she is friendly and always in touch with young people”.*

The intervention helped mitigate the risks faced by these young migrants through protective factors such as the peer supporters who acted as models to the young people, whom they were close to in age and had themselves been migrants to these communities.

## Discussion

We have described how young migrants in two towns in southwestern Uganda face sexual health risks in their first months, as they find ways to make money in the new place. We explain how a pilot intervention, co-designed with young people, provided a limited support structure for young migrants to help them access sexual and reproductive health care as well as give them someone like them to talk to. The intervention thus served to mitigate some of the risks the young people faced.

Being economically insecure pre-disposed the young people in our study to risk. From a gendered perspective, some of the young women engaged in transactional sex with multiple clients to supplement the little income earned and this heightened their risk to unwanted pregnancies, unsafe abortion and STI and HIV infections as has been similarly noted in other studies (7, 11, 25, 26). Consequently, the immediate risk faced by the women meant that they had to engage with SRH services quickly as they needed treatment. However, structural barriers such as long waiting times, none or few health care workers and lack of drugs (14) plus lack of money to afford private services often kept them away from health services which meant they remained untreated and yet continued engaging in sexual behaviour with multiple partners thus contributing to re-infections and new infections in the population.

On the other hand, young men were often involved in jobs that kept them occupied and they remained busy all time, sometimes in places located far away from the centre of town. This was a protective factor in the sense that they did not encounter sexual risk as they worked, a finding in agreement with other studies (18, 23) about social protection for harm reduction. However, once they gained a more substantial income from their jobs, many engaged in risky sexual behaviour. Previous studies have documented that men delay to seek treatment, often driven by social constructions of masculinity (27, 28). The “lending a hand” intervention demonstrated that men could utilize SRH services once taken closer to them as has been noted elsewhere (29, 30). Therefore, devising the means to extend SRH services to locations closer to where men work may increase their engagement with the services.

Prior research shows that young people mainly migrate to seek for employment or education (2, 3, 31, 32) and this becomes their primary focus putting other needs, including their health, second. Coupled with this, migrants face unique barriers to accessing health care including the inability to speak the native language and lack of knowledge about existing services (10, 13, 17, 33). As a result, they are likely to be less pro-active in accessing health services even when they are within their reach. Therefore, to increase the utilisation of SRH services among this population, interventions must be tailored to the needs of migrants. Providing support with the language skills to help with access to health facilities and the delivery of services through people (models) they can identify with, supports access. In that way, it creates a form of trust for the young people that they are speaking to people who they can easily identify with (13).

Despite the apparent vulnerability to risk, many young people are reluctant to go for SRH services due to the stigma associated with accessing these services in known public facilities. Many for instance, feared that they would be labelled promiscuous, and HIV positive (14, 34). In our study, some could not go to access condoms even when they knew where they can be got from. Through the peer supporters, they received condoms at their convenience, a service mentioned as being of value elsewhere (13). What we learn from the young people in this study is that it is not enough to provide the services, but also important that some of the structural barriers, like stigma associated with access to these services are addressed through innovative ways such as the creativity that came with this intervention where young people picked condoms from the peer supporters homes as opposed to going to health facilities.

We saw that the resilience of some young people was increased when protective factors were put in place and this improved access and use of SRH services as documented elsewhere (30). For instance, with increased knowledge about availability of services, young people can reach out and utilise the services (13) and young women who engaged in sex work went to the health facility and received PrEP which they mentioned that it benefited them in the sense that they were less fearful about having sex with men who did not want to use condoms but were offering them a lot of money, which they found hard to refuse (35).

## Limitations and strengths of the study

A limitation of the study was the loss to follow-up among our participants which meant that we interviewed fewer participants at the second round. As a result, we did not get an opportunity to learn about the reasons for onward migration and how they managed their vulnerability to new risky situations. Another limitation in our study was that we were unable to assess the sustainability of access to these services, after the conclusion of our study. Given the results presented in this paper are from a small number of young people in only two small towns in one part of Uganda our findings may not be generalisable to the experiences of all young migrants in these settings and elsewhere in Uganda.

The strength of this study was in being able to provide insights into the gendered risks among young migrants and testing out that protective factors, if put in place, can improve access to and utilisation of SRH services. By interviewing a young person twice at different time points, we were able to assess how these migrants navigated risk from early on when they arrived and the changes that the intervention introduced them to cope with the risk, including the resilience that was exhibited by some who devised ways to manage their risk, once provided with information from the intervention. Adapting the implementation of this intervention from being purely a physical one to a virtual one as well, when we set up a call centre to enable young migrants call into the intervention during the COVID-19 pandemic contributed to improved access to and utilisation of SRH services for this population through referral mechanisms. Therefore, the lessons learnt from this intervention warrant further consideration.

## Conclusion

Young migrants encounter heightened sexual health risks very early when they move to their host communities and are trying to settle in. The intervention helped to address the structural factors such as stigma and the long waiting hours at the facilities that impeded access to SRH services. Providing an enabling environment (role-models, social factors) for young migrants will support them to navigate risky situations as they adapt to the new host environments.

We noted gendered differences in risk as females tended to engage in risky sexual behaviours much earlier in their youth, compared to males by using sex work to earn an income. However, both males and females faced similar barriers in accessing SRH services. Finally, peer supporters managed to recruit young people of both genders into the intervention. However, we noticed that the male peers preferred to immediately refer all female migrants to the study counsellor for advice, while they were prepared to initially handle concerns of fellow male peers before referring them for further help. This could be due to societal norms which shape gendered interactions, with the young men feeling more comfortable giving advice to other males but fearing being misinterpreted or misunderstood by either the females themselves, or members from the wider community. The female peers were more experienced and had stayed longer in the community and, as a result, exhibited confidence that made both male and female peers comfortable in seeking advice from them. To increase the use of SRH services among this population, interventions need to put in the context of their gendered needs.

## Recommendation

We recommend that as a first step to increasing access and utilization of SRH services among migrant populations, outreach services can be provided for this population. For instance, the government and non-governmental organisations could set up drop in centres within the community, equipped with support services to provide health checks and referral services. Some of the staff could be one or two young people who speak languages which may be familiar to migrants, to help them navigate the services.

## Data Availability

The data supporting the conclusions of this article may be made available on reasonable request through the corresponding author
